# A Prolonged Case of Postpartum Psychosis: Challenges and Management

**DOI:** 10.1192/j.eurpsy.2026.11218

**Published:** 2026-07-31

**Authors:** C. Aksoy Poyraz, N. Boyraz, M. M. Kırpınar, N. G. Usta Sağlam

**Affiliations:** Psychiatry, İstanbul University-Cerrahpaşa, İstanbul, Türkiye

## Abstract

**Introduction:**

Postpartum psychosis (PPP) is commonly indicative of the need for immediate inpatient psychiatric treatment. The clinical presentation is marked by an abrupt onset of intense affective and psychotic symptoms and a fluctuating trajectory.

**Objectives:**

A patient with a prior episode of PPP experienced rapid psychiatric deterioration within one month following the delivery of her second child. The extended and treatment-resistant course of the episode emphasizes the necessity of prompt treatment initiation following childbirth, especially in individuals with a history of postpartum psychosis.

**Methods:**

Mental state examination, family interview, Magnetic resonance imaging (MRI) of the brain, hematologic, serologic and biochemical tests were performed.

**Results:**

A 30-year-old woman was admitted to the psychiatry service of the hospital 30 days after the birth of her second child with symptoms of confusion, agitation and delusions. Olanzapine 20 mg/day and valproate 1000 mg/day were initiated. On the 7th day of hospitalization, olanzapine was switched to risperidone 6 mg/day. Shortly, electroconvulsive therapy (ECT) was commenced due to inadequate response, with a total of six sessions. Following significant clinical improvement, the patient’s treatment regimen included lithium 900 mg/day, risperidone 6 mg/day, she was discharged on day 25. After 27 days post-discharge, she was rehospitalised due to the recurrence. ECT was initiated again, risperidone was discontinued, clozapine was initiated 50 mg/day and increased to 200 mg/day, along with amisulpride 800 mg/day. She improved and was discharged with the addition of lithium 1200 mg/day. After 37 days of discharge due to recurrence she was readmitted to the psychiatry ward clozapine was increaed to 350 mg/day and shortly was discharged because she had COVID-19 infection. Over the next 2 months, she experienced partial remission with fluctuating psychotic symptoms. Following the cessation of clozapine due to low tolerability, the patient underwent a series of 5 ECT sessions and shortly remission was achieved.

**Conclusions:**

A history of bipolar disorder and/or prior episodes of PPP are the primary risk factors for PPP development. Prompt treatment initiation following childbirth is imperative to address potentially treatment-resistant cases that may persist for months. This case despite being hospitalized three times and receiving intensive treatment, including lithium, high doses of antipsychotics, and the rarely used clozapine for postpartum psychosis, did not achieve remission. Our case underscores the distinctive significance of ECT in these situations. While ECT is typically viewed as a final recourse for treatment-resistant PPP, its implementation earlier in the treatment process and the possibility of extended administrations should be contemplated to ensure complete remission, provided that the treatment is well-tolerated.

**Disclosure of Interest:**

None Declared

